# Supplementary material for: Association Between Decision-Making Styles, Personality Traits, and Socio-Demographic Factors in Women Choosing Voluntary Pregnancy Termination: A Cross-Sectional Study
Source: Eur J Investig Health Psychol Educ. 2025 Oct 16;15(10):214. doi: 10.3390/ejihpe15100214 (PMC12563433; doi:10.3390/ejihpe15100214)
Supplement: Supplementary file 1 [file ejihpe-15-00214-s001.zip › ejihpe-3821912-supplementary.pdf]

Association Between Decision-Making Styles, Personality Traits, and Socio-Demographic Factors in Women Choosing Voluntary Pregnancy Termination: A Cross-Sectional Study

Letizia Lorusso, Nicola Bartolomeo, Maria Elvira Metta, Daphne Gasparre, Patrizia Pignataro, Giulia Caradonna, Paolo Taurisano, Paolo Trerotoli

Supplementary materials

Supplementary Results

In Table S1, when comparing the age groups (< 30 years vs. ≥ 30 years) on the trait "Agreeableness" in a multivariable analysis (i.e., controlling for covariates such as education, parity, and marital status), the estimated adjusted difference between the groups is –0.8 (95% CI: –5.0 to 3.4), suggesting that, net of statistically significant covariates, there is no evidence of a meaningful difference in "Agreeableness" between age groups. The same results for the estimated adjusted difference in parity 2.0 (95% CI: –2.0 to 5.9), and marital status 0.1 (95% CI: –3.7 to 3.9). In that same multivariable model, education is significantly associated with "Agreeableness": for instance, women with low educational attainment showed an adjusted mean of 75.7 (95% CI: 72.2–79.2), compared to 83.8 (95% CI: 80.6–87.1) for those with high education — a mean difference of –8.2 (95% CI: –13.9 to –2.4), suggesting a positive association between high education and "Agreeableness" when controlling for age, parity, and marital status.

When comparing educational levels on the adjusted mean of the "Openness" dimension in a multivariable analysis controlling only for employment status and educational attainment, the adjusted mean for the "Openness" trait in the low and high education groups were 76.6 (95% CI: 72.3–80.9) and 84.8 (95% CI: 80.8–88.7), respectively, and the estimated adjusted difference between low and high education was –8.2 (95% CI: –15.4 to –0.9), indicating a positive association between high education and "Openness to Experience." The score of "Conscientiousness" and "Emotional Stability" dimensions was not significantly associated with any socio-demographic characteristic. The adjusted means for the "Extraversion" dimension, in a multivariable analysis controlling for employment status and educational level, were significantly higher among employed women (77.3; 95% CI: 74.8–79.8) than unemployed women (73.6; 95% CI: 70.8–76.4), with an estimated adjusted difference of 3.7 (95% CI: 0.04–7.4).**Table S1. Estimated least squares means from multivariable analyses examining differences in Big Five personality traits across socio-demographic groups.**

Socio-demographics characteristics	Agreeableness		Openness		Conscientiousness		Extraversion		Emotional Stability	
	Multivariable		Multivariable		Multivariable		Multivariable		Multivariable	
	LSMeans [95% CI]	Mean diff. [95% CI]	LSMeans [95% CI]	Mean diff. [95% CI]	LSMeans [95% CI]	Mean diff. [95% CI]	LSMeans [95% CI]	Mean diff. [95% CI]	LSMeans [95% CI]	Mean diff. [95% CI]
**Age**										
< 30 years	79.6 [76.4;82.7]	-0.8 [-5.0;3.4]							63.9 [59.2;68.6]	-2.6 [-9.2;4.0]
≥ 30 years	80.4 [78.1;82.6]								66.6 [63.1;70]	
**Employment status**										
Employed			80.6 [78;83.3]	0.5 [-3.5;4.4]			77.3 [74.8;79.8]	3.7 [0.04;7.4]		
Unemployed			80.2 [77.2;83.2]				73.6 [70.8;76.4]			
**Parity**										
With children	81.0 [78.3;83.6]	2.0 [-2.0;5.9]							66.5 [62.5;70.5]	2.5 [-3.6;8.6]
Without children	79.0 [76.4;81.6]								64 [60.1;67.9]	
**Education Status**										
Low	75.7 [72.2;79.2]	-4.7 [-9.4;0.1]*	76.6 [72.3;80.9]	-3.3 [-9.2;2.6]*	83.2 [78.8;87.6]	-0.2 [-6.2;5.8]*	73.1 [69.1;77.1]	-3.4 [-8.9;2.1]*		
Medium	80.4 [78.4;82.3]	-8.2 [-13.9;-2.4]**	79.9 [77.5;82.3]	-8.2 [-15.4;-0.9]**	83.4 [81;85.9]	-4.9 [-11.9;2.1]**	76.5 [74.2;78.7]	-3.8 [-10.5;3.0]**		
High	83.8 [80.6;87.1]	-3.5 [-7.9;0.9]***	84.8 [80.8;88.7]	-4.9 [-10.5;0.7]***	88.1 [84.3;91.9]	-4.7 [-10.1;0.7]***	76.9 [73.2;80.6]	-0.4 [-5.6;4.8]**		
**Marital Status**										
Not in a couple	80.0 [77.7;82.3]	0.1 [-3.7;3.9]							64.4 [61;67.8]	-1.6 [-7.7;4.4]
In a couple	79.9 [77.1;82.8]								66.1 [61.7;70.4]	

Legend: *Low vs. Medium; **Low vs. High; ***Medium vs. High; LSMeans: Adjusted means estimated from the model, controlling for other variables.

In Table S2 when we compared the adjusted means of the traits “Negative affect”, “Antagonism”, “Disinhibition”, “Detachment” and “Psychoticism” in the multivariable model controlling for age groups, employment status, education, parity and marital status: the score of “Disinhibition” and “Antagonism” dimensions were not significantly associated with any socio-demographic characteristic. In the multivariable analysis comparing educational levels, adjusted means of the “Detachment” and “Psychoticism” dimensions were examined while controlling for age group, employment status, parity (excluded only for “Detachment”), and marital status. The adjusted means of “Detachment” were significantly lower among women with higher educational attainment compared to those with low (mean: 5.2; 95% CI: 4.0–6.5) and medium (mean: 4.6; 95% CI: 3.9–5.3) levels of education. The estimated adjusted differences were 2.3 (95% CI: 0.2–4.3) for low vs. high education, and 1.6 (95% CI: 0.05–3.2) for medium vs. high education. The adjusted means for

"Psychoticism" were significantly higher for women with medium education 5.0 (95% CI: 4.2–5.8) compared to those with higher education 3.1 (95% CI: 1.8–4.5), with an adjusted mean difference of 1.9 (95% CI: 0.1–3.8). The adjusted mean for the dimension "Negative affect" was significantly lower for women with children 5.9 (95% CI: 4.9–6.9) compared to those for women without children 7.5 (95% CI: 6.6–8.5) with an adjusted mean difference of -1.6 (95% CI: -3.1 to -0.2).

Table S2. Estimated least squares means from multivariable analyses examining differences in PID-5 personality traits across socio-demographic groups.

**Socio-demographics characteristics**	**Negative affect**		**Antagonism**		**Disinhibition**		**Detachment**		**Psychoticism**	
	Multivariable		Multivariable		Multivariable		Multivariable		Multivariable	
	LSMeans [95% CI]	Mean diff. [95% CI]	LSMeans [95% CI]	Mean diff. [95% CI]	LSMeans [95% CI]	Mean diff. [95% CI]	LSMeans [95% CI]	Mean diff. [95% CI]	LSMeans [95% CI]	Mean diff. [95% CI]
**Age**										
< 30 years	6.6 [5.4;7.7]	-0.3 [-1.8;1.2]			5.3 [4.1;6.4]	0.5 [-1;2]	4.4 [3.3;5.5]	0.3 [-1.1;1.6]	4.7 [3.5;6]	0.5 [-1.2;2.3]
≥ 30 years	6.9 [6.0;7.7]				4.8 [4;5.6]		4.1 [3.4;4.9]		4.2 [3.3;5.1]	
**Employment status**										
Employed			2.6 [1.9;3.3]	0.5 [-1.6;0.5]			4.1 [3.3;4.9]	-0.3 [-1.4;0.9]	4.4 [3.4;5.3]	-0.2 [-1.5;1.1]
Unemployed			3.2 [2.3;4.0]				4.4 [3.5;5.3]		4.6 [3.6;5.6]	
**Parity**										
With children	5.9 [4.9;6.9]	-1.6 [-3.1;-0.2]							3.9 [2.9;5]	-1 [-2.6;0.6]
Without children	7.5 [6.6;8.5]								5.0 [3.9;6]	
**Education Status**										
Low	6.9 [5.5;8.2]	-0.5 [-2.3;1.4]*	3.3 [2.1;4.4]	0.2 [-1.4;1.8]*	6 [4.6;7.4]	0.7 [-1.1;2.6]*	5.2 [4;6.5]	0.6 [-1;2.3]*	5.3 [3.8;6.7]	0.2 [-1.7;2.2]*
Medium	7.4 [6.6;8.1]	0.9 [-1.3;3.1]**	3.1 [2.4;3.7]	1 [-1.0;2.9]**	5.3 [4.5;6]	2.2 [0.;4.4]**	4.6 [3.9;5.3]	2.3 [0.2;4.3]**	5 [4.2;5.8]	2.1 [-0.3;4.6]**
High	5.9 [4.7;7.2]	1.4 [-0.3;3.2]***	2.3 [1.2;3.4]	0.8 [-0.7;2.3]***	3.8 [2.6;5]	1.5 [-0.2;3.2]***	2.9 [1.8;4.1]	1.6 [0.05;3.2]***	3.1 [1.8;4.5]	1.9 [0.1;3.8]***
**Marital Status**										
Not in a couple					5.3 [4.4;6.1]	0.5 [-1;1.9]	4.4 [3.6;5.1]	0.2 [-1.1;1.5]	4.4 [3.5;5.4]	-0.1 [-1.6;1.5]
In a couple					4.8 [3.7;5.9]		4.1 [3.2;5.1]		4.5 [3.3;5.7]	

Legend: *Low vs. Medium; **Low vs. High; ***Medium vs. High; LSMeans: Adjusted means estimated from the model, controlling for other variables.

There were no associations between any of the GDMS domains and socio-demographic characteristics, except for the dimensions "Avoidant" and "Intuitive", for which the scores were higher for women with low education levels respect them with high educational attainment. In the univariate analysis, a slightly statistically significant increase in the

“Avoidant” score was observed for all the PID-5 dimensions. Although the results initially appeared statistically significant, this significance disappeared in multivariable analysis. It can be hypothesized that, in this group of women, the very high “Conscientiousness” scores account for most of the variance. Overall, “Conscientiousness” levels were remarkably high, indicating that the women in the sample may be highly conscientious. While “Avoidant” scores tend to increase in line with all PID-5 domain scores, in our sample, PID-5 scores were generally low and therefore contributed minimally to the multivariable model. In our sample, women with higher score in “Conscientiousness” tended to show lower “Avoidant” scores. The decreases in all the PID-5 dimensions were correlated with an increase in the “Rational” scores, in the univariate analysis. In the multivariable model a decrease statistically significant in the score of “Disinhibition” dimension and an increase in “Conscientiousness” was associated with an increase in a “Rational” decision style. In the univariate analysis, a statistically significant increase in the “Spontaneous” score was associated with an increase in all the PID-5 dimensions. In the multivariable model, a statistically significant increase in the scores for the BFQ dimension “Extraversion” was associated with an increase in the “Spontaneous” decision-making style, but with a statistically significant decrease in the scores for the BFQ dimensions “Conscientiousness” and “Emotional Stability”.

Table S3. Results of the univariate and Multivariable analysis comparing GDMS score and socio-demographic characteristics, BFQ, and PID-5 scores.

Socio-demographics characteristics, BFQ and PID-5 dimensions	Dependent				Avoidant				Intuitive				Rational				Spontaneous			
	Univariate		Multivariable		Univariate		Multivariable		Univariate		Multivariable		Univariate		Multivariable		Univariate		Multivariable	
	Beta (SE)	*p*	Beta (SE)	*p*	Beta (SE)	*p*	Beta (SE)	*p*	Beta (SE)	*p*	Beta (SE)	*p*	Beta (SE)	*p*	Beta (SE)	*p*	Beta (SE)	*p*	Beta (SE)	*p*
**Age**																				
< 30 years vs. ≥ 30 years	-0.2 (0.8)	0.841			-0.1 (0.8)	0.929			-0.3 (0.6)	0.586			0.44 (0.8)	0.569			0.4 (0.8)	0.654		
**Employment status**																				
Employed vs Unemployed	-0.5 (0.8)	0.573			-1.1 (0.8)	0.201			-0.3 (0.6)	0.604			-0.9 (0.8)	0.911			0.1 (0.8)	0.921		
**Parity**																				
With children vs. Without children	0.2 (0.8)	0.847			0.2 (0.8)	0.804			0.4 (0.6)	0.513			-0.6 (0.8)	0.445			-0.2 (0.8)	0.854		
**Educational Level**																				
Low vs High	-0.2 (1.3)	0.968			2.5 (1.3)	0.130	1.2 (1.3)	0.615	2.0 (0.9)	**0.044**	1.8 (0.9)	0.136	-0.5 (1.2)	0.586			0.6 (1.3)	0.895		
Medium vs High	0.1 (1.0)				1.1 (1.0)		-0.4 (1.0)	0.900	0.1 (0.7)		-0.2 (0.7)	0.955	-0.9 (0.9)				0.3 (1.0)			
**Marital Status**																				

Not in a couple vs. In a couple	0.6 (0.8)	0.763			-0.2 (0.8)	0.852			-0.6 (0.6)	0.358			0.01 (0.8)	0.985			-0.1 (0.8)	0.950		
**Big-Five Dimensions (BFQ)**																				
Agreeableness	0.01 (0.05)	0.887			-0.1 (0.05)	**0.044**	0.06 (0.05)	0.259	0.02 (0.04)	0.605			0.1 (0.04)	0.212	-0.04 (0.04)	0.364	-0.1 (0.1)	0.182	0.01 (0.05)	0.840
Openness	-0.02 (0.04)	0.531			-0.07 (0.04)	0.090	0.04 (0.04)	0.343	-0.01 (0.03)	0.711			0.1 (0.04)	**0.046**	0.01 (0.03)	0.732	-0.04 (0.04)	0.323		
Conscientiousness	-0.02 (0.04)	0.654			-0.18 (0.04)	**<0.001**	-0.16 (0.04)	**<0.001**	-0.04 (0.03)	0.227	-0.03 (0.03)	0.364	0.2 (0.03)	**<0.001**	0.14 (0.04)	**<0.001**	-0.1 (0.04)	**<0.001**	-0.1 (0.04)	**0.003**
Extraversion	-0.08 (0.04)	0.077	-0.07 (0.04)	0.110	-0.1 (0.04)	**0.014**	-0.03 (0.05)	0.540	0.01 (0.03)	0.841			-0.02 (0.04)	0.637			0.1 (0.04)	**0.005**	0.2 (0.04)	**<0.001**
Emotional Stability	-0.04 (0.03)	0.181	-0.03 (0.03)	0.270	-0.09 (0.03)	**0.004**	-0.07 (0.03)	**0.039**	-0.02 (0.02)	0.410			0.05 (0.03)	0.073	0.02 (0.03)	0.534	-0.1 (0.03)	**0.040**	-0.1 (0.03)	**0.032**
**PID-5 Dimensions**																				
Negative affect	0.11 (0.13)	0.639			0.41 (0.12)	**<0.001**	0.11 (0.16)	0.487	0.11 (0.09)	0.247	0.11 (0.09)	0.247	-0.1 (0.1)	0.515			0.3 (0.1)	**0.006**	0.0 (0.2)	0.996
Antagonism	-0.1 (0.15)	0.519			0.44 (0.14)	**0.003**	0.12 (0.17)	0.473	-0.02 (0.11)	0.865			-0.3 (0.1)	**0.047**	0.02 (0.15)	0.888	0.5 (0.1)	**<0.001**	0.3 (0.2)	0.062
Disinhibition	-0.14 (0.13)	0.633			0.48 (0.12)	**<0.001**	-0.06 (0.17)	0.749	0.07 (0.09)	0.451			-0.61 (0.1)	**<0.0001**	-0.58 (0.14)	**<0.0001**	0.54 (0.12)	**<0.001**	0.11 (0.17)	0.500
Detachment	0.1 (0.14)	0.575			0.53 (0.13)	**<0.001**	0.19 (0.2)	0.356	0.02 (0.1)	0.823			-0.2 (0.12)	0.115	0.28 (0.15)	0.075	0.3 (0.13)	**0.026**	0.09 (0.2)	0.649
Psychoticism	-0.07 (0.12)	0.793			0.45 (0.11)	**<0.001**	0.13 (0.16)	0.433	0.08 (0.09)	0.394			-0.25 (0.11)	**0.028**	0.05 (0.14)	0.728	0.31 (0.12)	**0.010**	-0.01 (0.16)	0.959

Legend: SE: Standard Error

To assess potential multicollinearity among the predictor variables in each model—where the dependent variables were the Dependent, Avoidant, Intuitive, Rational, and Spontaneous domains of the GDMS test—Variance Inflation Factor (VIF) and tolerance values were examined across multiple Multivariable models. VIF values ranged from 1.02 to 2.83, while tolerance values ranged from 0.35 to 0.98. All VIF values remained well below the commonly accepted threshold of 5, and all tolerance values were above the critical value of 0.20. These results indicate no evidence of problematic multicollinearity among the variables. Notably, the Dependent and Intuitive dimensions showed the lowest VIFs values (1.02 and 1.03, respectively), suggesting minimal redundancy with other predictors, while Spontaneous and Avoidant exhibited slightly higher VIFs (2.82 and 2.83, respectively), still within acceptable limits (Table S4).

Table S4. Multicollinearity Diagnostics for Personality Dimensions.

BFQ and PID-5 dimensions	General Decision-Making Style (GDMS) domains				
	Dependent	Avoidant	Intuitive	Rational	Spontaneous
	Multivariable VIF (tolerance)	Multivariable VIF (tolerance)	Multivariable VIF (tolerance)	Multivariable VIF (tolerance)	Multivariable VIF (tolerance)
**Big-Five Dimensions**					
Agreeableness		1.52 (0.66)		1.51 (0.66)	1.43 (0.70)
Openness		1.47 (0.68)		1.39 (0.72)	
Conscientiousness		1.55 (0.65)	1.03 (0.97)	1.47 (0.68)	1.52 (0.66)
Extraversion	1.02 (0.98)	1.45 (0.69)			1.37 (0.73)
Emotional Stability	1.02 (0.98)	1.43 (0.70)		1.17 (0.86)	1.40 (0.71)
**PID-5 Dimensions**					
Negative affect		2.03 (0.49)	1.03 (0.97)		2.00 (0.50)
Antagonism		1.77 (0.57)		2.24 (0.45)	1.76 (0.57)
Disinhibition		2.41 (0.41)		2.20 (0.45)	2.40 (0.42)
Detachment		2.83 (0.35)		2.24 (0.45)	2.82 (0.35)
Psychoticism		2.22 (0.45)		2.15 (0.46)	2.21 (0.45)
